# Advances in pathogenesis and treatment of vascular endothelial injury-related diseases mediated by mitochondrial abnormality

**DOI:** 10.3389/fphar.2024.1422686

**Published:** 2024-08-30

**Authors:** Boxian Pang, Guangtong Dong, Tieliang Pang, Xinyao Sun, Xin Liu, Yifeng Nie, Xing Chang

**Affiliations:** ^1^ Graduate School, Beijing University of Chinese Medicine, Beijing, China; ^2^ CAS Center for Excellence in Nanoscience, National Center for Nanoscience and Technology, Beijing, China; ^3^ Beijing Anding Hospital, Capital Medical University, Beijing, China; ^4^ Bioscience Department, University of Nottingham, Nottingham, United Kingdom; ^5^ Guang’anmen Hospital, China Academy of Chinese Medical Sciences, Beijing, China

**Keywords:** mitochondria abnormality, endothelial cell injury, cardiovascular diseases, ischemic stroke, Alzheimer’s disease, treatment

## Abstract

Vascular endothelial cells, serving as a barrier between blood and the arterial wall, play a crucial role in the early stages of the development of atherosclerosis, cardiovascular diseases (CVDs), and Alzheimer’s disease (AD). Mitochondria, known as the powerhouses of the cell, are not only involved in energy production but also regulate key biological processes in vascular endothelial cells, including redox signaling, cellular aging, calcium homeostasis, angiogenesis, apoptosis, and inflammatory responses. The mitochondrial quality control (MQC) system is essential for maintaining mitochondrial homeostasis. Current research indicates that mitochondrial dysfunction is a significant driver of endothelial injury and CVDs. This article provides a comprehensive overview of the causes of endothelial injury in CVDs, ischemic stroke in cerebrovascular diseases, and AD, elucidating the roles and mechanisms of mitochondria in these conditions, and aims to develop more effective therapeutic strategies. Additionally, the article offers treatment strategies for cardiovascular and cerebrovascular diseases, including the use of clinical drugs, antioxidants, stem cell therapy, and specific polyphenols, providing new insights and methods for the clinical diagnosis and treatment of related vascular injuries to improve patient prognosis and quality of life. Future research should delve deeper into the molecular and mechanistic links between mitochondrial abnormalities and endothelial injury, and explore how to regulate mitochondrial function to prevent and treat CVDs.

## 1 Introduction

The endothelium is a single layer of cells composed of endothelial cells, serving as a uniform passive barrier between circulating blood and the underlying arterial wall (Florey, 1966). This special layer of cells acts as an important protective barrier, separating and safeguarding the underlying tissue from the dynamic environment of the circulating blood. While providing this essential barrier, the endothelium also plays an indispensable role in regulating vascular tone by releasing various substances that dilate or constrict blood vessels. Additionally, the endothelium maintains the structural integrity of blood vessels by producing extracellular matrix components and regulating cell adhesion and proliferation. Due to their position directly in contact with the blood, endothelial cells are aptly referred to as the “frontline” cells in combating vascular diseases (Davidson and Duchen, 2007). This unique position exposes them to various circulating elements, including nutrients, oxygen, hormones, and waste products. However, it also makes them particularly susceptible to harmful molecules such as vascular endothelial growth factors, inflammatory cytokines, and reactive oxygen species (ROS). These harmful factors can trigger endothelial dysfunction, a key early event in the development of atherosclerosis and other cardiovascular diseases (CVDs) (Chrysohoou et al., 2018). The frontline position of endothelial cells not only makes them prone to damage but also places them in an ideal position to act as “scouts,” constantly monitoring the vascular environment (Davidson and Duchen, 2007). Moreover, these cells are equipped with various sensors and receptors capable of detecting changes in blood flow, pressure, and biochemical composition of the blood (Winegrad, 1997). Once adverse conditions are detected, endothelial cells can initiate a series of protective responses, including the secretion of anti-inflammatory molecules, regulation of coagulation, and activation of repair mechanisms to restore vascular homeostasis (Davidson and Duchen, 2007).

**FIGURE 1:**
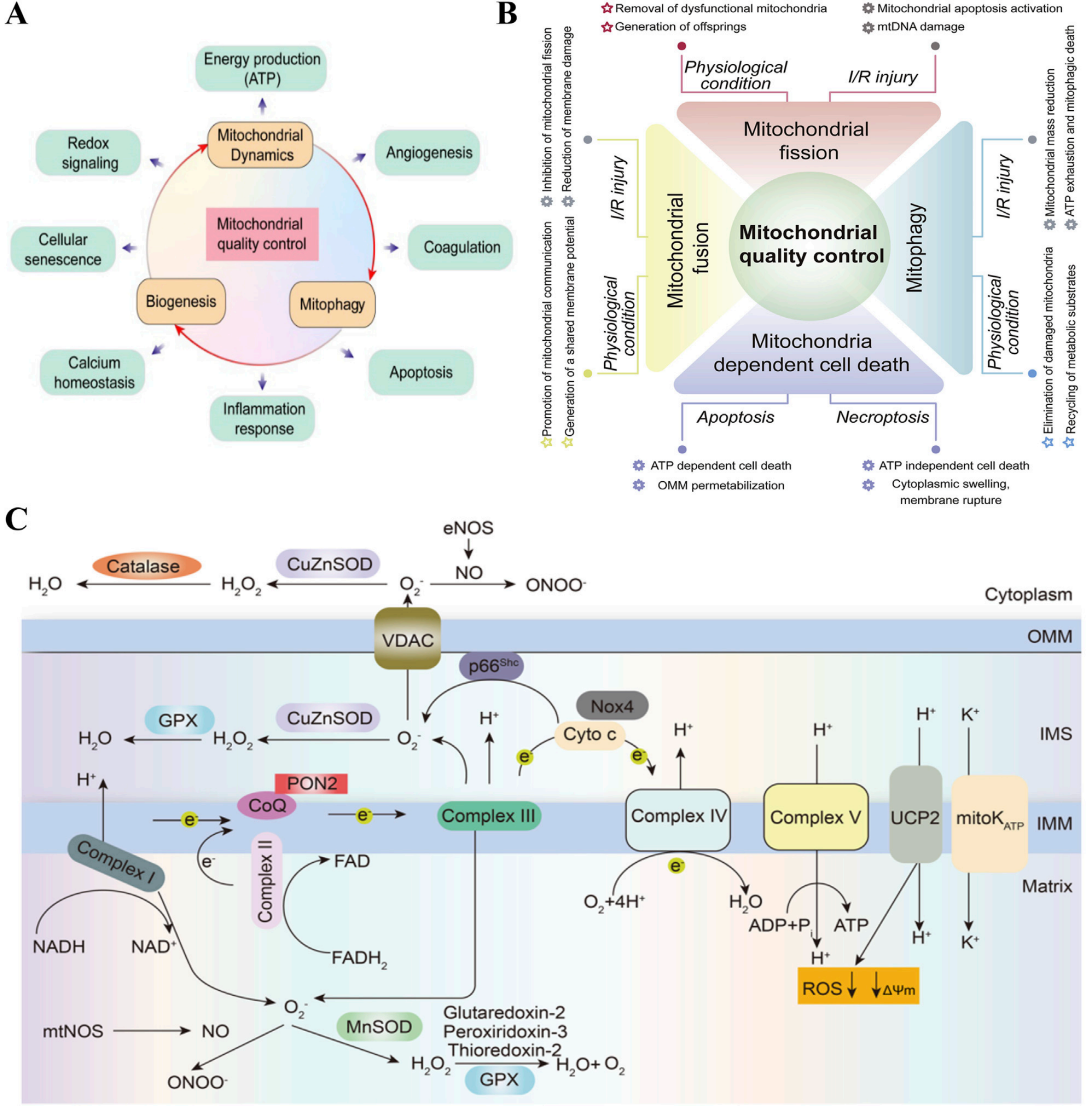
Mitochondria affect endothelial cell function and metabolism. **(A)** Mitochondria regulate several key biological processes in the endothelial cells, such as energy production, angiogenesis, coagulation, apoptosis, inflammation response, calcium homeostasis, cellular senescence and redox signaling. Copyright 2021, Reprinted with from permission from Ivyspring International Publisher (Chang et al., 2021). **(B)** MQC coordinates various processes including fusion, division, mitotic phagocytosis and mitochondria-controlled cell death under physiological conditions and I/R injury. Mitochondrial dysfunction in quality-control processes is the main mechanism of cardiac I/R injury. Copyright 2020, Reprinted with from permission from Elsevier B.V. (Wang and Zhou, 2020). **(C)** Overview of mitochondrial metabolism in endothelial cells. In endothelial cells, the mitochondrial respiratory chain complexes I-IV create a proton gradient across the inner mitochondrial membrane (IMM), which powers adenosine triphosphate (ATP) production by ATP synthase (Complex V). Electrons from NADH and FADH2 flow through Complexes I and II, then to Complex III via ubiquinol (CoQ). Electrons are passed from Complex III to Complex IV by cytochrome c, reducing oxygen to water. This electron flow is coupled with proton (H^+^) transfer, creating an electrochemical gradient (ψm). Protons re-enter the matrix through Complex V to produce ATP, and uncoupling proteins and mitoK ATP channels allow protons to bypass this process, reducing reactive oxygen species (ROS) formation. PON2: paraoxonase 2, NOX4: nicotinamide adenine dinucleotide phosphate oxidase 4, UCP2: uncoupling protein 2. Copyright 2022, Reprinted with from permission from Nature Publishing Group (Sun et al., 2022).

Mitochondria, known as the powerhouses of the cell, are the primary sites of cellular aerobic respiration. They meet the cell’s energy needs by producing adenosine triphosphate (ATP) using oxygen and glucose. In addition to energy production, mitochondria regulate several key biological processes in endothelial cells, including redox signaling, cell aging, calcium homeostasis, angiogenesis, apoptosis, and inflammatory responses (Figure 1A) (Chang et al., 2021). Furthermore, mitochondria in endothelial cells are considered central oxygen sensors in the vascular system, acting as local environmental sensors (Chang et al., 2021). These multifaceted roles largely depend on the mitochondrial quality control (MQC) system (Figure 1B), which includes mitochondrial dynamics (fusion and fission), mitophagy, and mitochondrial biogenesis (Wang and Zhou, 2020; Wang et al., 2020c; Wang et al., 2020b). The MQC system is an integrated network that monitors mitochondrial quality and serves as an endogenous cell protection mechanism, crucial for maintaining mitochondrial homeostasis and function (Gan et al., 2018). The state of mitochondrial homeostasis is a determining factor in whether endothelial cells are normal or damaged, thus highlighting the importance of mitochondria in vascular health (Figure 1C) (Pang et al., 2024).

Currently, CVDs account for approximately one-third of all deaths worldwide (Joseph et al., 2017). Among these CVDs, atherosclerotic cardiovascular disease (ASCVD), including ischemic heart disease (IHD) and ischemic stroke, is increasingly viewed as a type of cardiovascular disease in both Chinese and international cardiovascular disease prevention and treatment guidelines due to its shared pathology, common risk factors, and unified primary and secondary prevention strategies (Zhao et al., 2019). A meta-analysis by Xie et al. suggests that atherosclerosis is closely related to the onset and progression of Alzheimer’s disease (AD) (Xie et al., 2020).

Atherosclerosis, as a vascular pathological manifestation, may play a crucial role in the pathogenesis of AD by reducing cerebral blood flow and affecting Amyloid-β (Aβ) clearance (Gupta and Iadecola, 2015). Simultaneously, growing evidence indicates that risk factors for atherosclerosis have also become significant triggers for AD, as vascular risk factors can accelerate the progression of AD by promoting the accumulation of beta-amyloid protein in the brain (Li et al., 2010; Saeed et al., 2023). Furthermore, on a molecular mechanism level, it has been demonstrated that C/EBPβ and asparagine endopeptidase (AEP) play important roles in the pathogenesis of both Atherosclerosis and AD. The C/EBPβ/AEP signaling pathway couples atherosclerosis with AD by mediating vascular pathology (Liao et al., 2022). All of the above underscores the urgency and importance of exploring the underlying mechanisms of cardiovascular and cerebrovascular diseases.

This paper provides a comprehensive review of the pathogenesis of endothelial damage in cardiovascular diseases and cerebrovascular diseases (Ischemic stroke and Alzheimer’s disease). By thoroughly elucidating the roles and potential mechanisms of mitochondria in these diseases, it aims to develop more effective treatment strategies. Additionally, the review offers corresponding therapeutic strategies for cardiovascular and cerebrovascular diseases, including the use of clinical drugs such as medications, antioxidants, stem cell therapy, and specific polyphenols. It aims to provide new insights and treatment methods for the clinical diagnosis and treatment of related vascular injuries, serving as a reference for improving patient prognosis and quality of life in clinical practice.

## 2 Cardiovascular diseases

**FIGURE 2:**
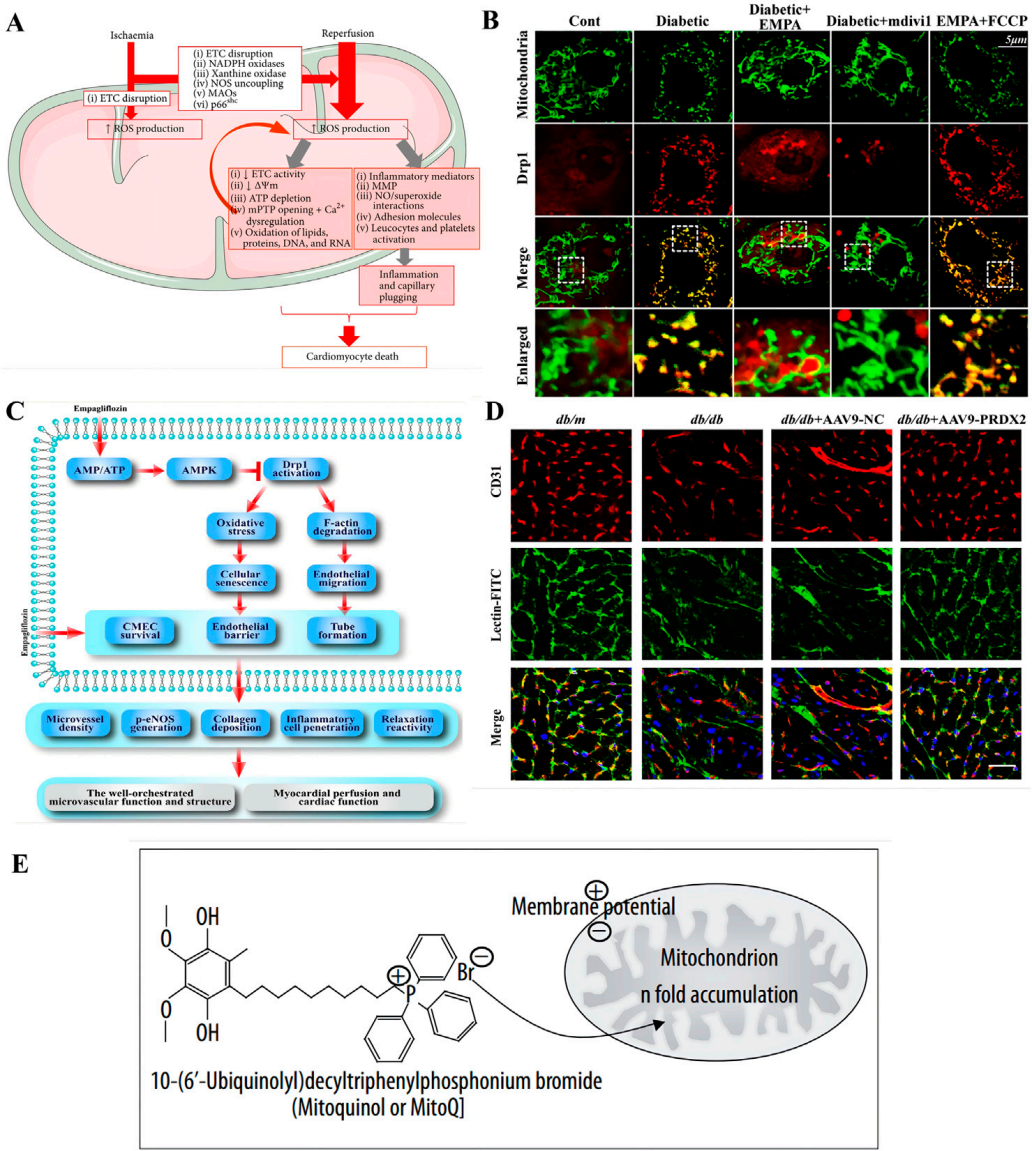
Pathogenesis and therapeutic strategies of cardiovascular diseases based on mitochondrial abnormality. **(A)** The role of mitochondrial reactive oxygen species (ROS) in ischemia-reperfusion (I/R) injury is multifaceted. When cells experience ischemia-induced hypoxia, it leads to a halt in the electron transport chain (ETC) within the inner mitochondrial membrane (IMM), which in turn triggers the production of ROS. The reoxygenation phase is further exacerbated by the heightened activity of monoamine oxidases (MAOs), NADPH oxidase, and p66shc, as well as by structural alterations in xanthine oxidase and the uncoupling of nitric oxide (NO) synthase, all of which contribute to an increase in ROS levels. Copyright 2016, Reprinted with from permission from Hindawi Publishing Group (Muntean et al., 2016). **(B,C)** Empagliflozin attenuates myocardial microvascular damage by mediating mitochondrial fission. **(B)** Empagliflozin diminishes diabetic-induced mitochondrial fragmentation and regulated the balance of proteins responsible for mitochondrial fission and fusion. Co-localization of Drp1 and mitochondria. The boxed area under each micrograph represents the amplifification of the white square. More Drp1 was located on fragmented mitochondria, while empagliflozin reduced Drp1 migration ontomitochondria. Copyright 2017, Reprinted with from permission from Elsevier B.V (Zhou et al., 2018). **(C)** The diagram illustrates the protective mechanisms by which empagliflozin shields the microvasculature from damage in diabetes. Empagliflozin initiates its protective effect by stimulating the AMPK signaling pathway, which is regulated by the balance of AMP to ATP ratios. Once activated, AMPK influences the posttranscriptional phosphorylation of Drp1 at specific sites, Ser616 and Ser637. The modification inhibits Drp1’s capacity to migrate to the mitochondria, thereby impairing mitochondrial fission. The resulting reduction in mitochondrial fission slows down cellular aging and maintains the integrity of the endothelial barrier and permeability by reducing the excess production of ROS. **(D)** Endothelial-specific overexpression of PRDX2 alleviated cardiac microvascular injury and improved cardiac function after long-term diabetes. Isoliquitigenin could inhibit mitochondrial iron toxicity through PRDX2-MFN2-ACS14 pathway, thus effectively protecting cardiac microvessels in diabetic patients. The cardiac microvascular density was indicated by the number of CD31-positive microvessels (green), and microvascular perfusion was indicated by the ratio of FITC-positive microvessels (green) to CD31-positive microvessels (red). Scale bars = 40 mm. Copyright 2023, Reprinted with from permission from American Diabetes Association (Chen et al., 2023). **(E)** Utilizing lipophilic cations to selectively deliver MitoQ towards mitochondria within cells to target mitochondria. The accumulation of lipophilic cations within the mitochondria is facilitated by the mitochondrial membrane potential. These cations can readily traverse lipid bilayers due to the delocalization of their positive charge over a broad area, which enhances their permeability. Copyright 2007, Reprinted with from permission from International Scientific Literature Inc. (Milagros Rocha and Victor, 2007).

Endothelial cells and cardiomyocytes are the main cellular components of the heart, interacting with each other through various cellular signaling molecules. In cases of endothelial cell injury, this interaction can lead to a vicious cycle, ultimately resulting in cardiomyocyte damage and death (Figure 2A). Cardiac endothelial dysfunction has been identified as a major cause of CVDs, closely associated with mitochondrial abnormalities in endothelial cells that regulate their own structure and function (Zhang et al., 2023).

Mitochondria are the powerhouses of cells, producing ATP through oxidative phosphorylation. In the heart, to ensure sufficient oxygen supply to cardiomyocytes, endothelial cells require only a small amount of oxygen. Furthermore, endothelial cells rely more on glycolysis for energy rather than oxidative phosphorylation (Chang et al., 2021). Consequently, the relative mitochondrial content in cardiac endothelial cells is lower than that in cardiomyocytes. Serving as the selective permeable interface between blood and myocardium, the coronary microcirculation primarily regulates blood distribution to meet the dynamic needs of cardiomyocytes (Escaned and Lerman, 2020). Diabetic patients often suffer from chest pain, but invasive coronary angiography frequently does not detect obstructive coronary artery disease, which is thought to be caused by coronary microvascular endothelial dysfunction (CMD) (Taqueti and Di Carli, 2018). In CMD, abnormal coronary endothelial cells function or structural damage impairs blood flow regulation, preventing it from adjusting to the changing oxygen demands of cardiomyocytes from rest to stress states (Sun et al., 2022). Mitochondrial dysfunction can induce oxidative stress, disrupt metabolism, and activate endothelial cell apoptosis, thus exacerbating the progression of diabetic coronary microvascular complications (Sun et al., 2022). The mitochondrial quality surveillance (MQS) system responds to stress by altering mitochondrial metabolism, dynamics (fission and fusion), mitophagy, and biogenesis. During these processes, high glucose levels increase ROS in a time-dependent manner in human umbilical vein endothelial cells (HUVECs), thereby inhibiting the AMP-activated protein kinase (AMPK) pathway and suppressing the transcription of Peroxisome proliferator-activated receptor gamma coactivator 1-α (PGC1α) (Sun et al., 2022). The corresponding treatment using metformin or 5-aminoimidazole-4-carboxamide ribonucleoside to stimulate AMPK can enhance PGC1α transcription, mitochondrial transcription factor A (TFAM) expression, and mitochondrial proliferation (Kukidome et al., 2006). Other antioxidant strategies, such as supplementing with resveratrol (Csiszar et al., 2009) or nicotinamide mononucleotide (Tarantini et al., 2019; Wang H. et al., 2020), have also been shown to upregulate manganese superoxide dismutase expression in endothelial cells in a dose-dependent manner, thereby reducing mitochondrial reactive oxygen species (mtROS) production and enhancing mitochondrial biogenesis (Csiszar et al., 2009; Ungvari et al., 2009). Additionally, chicoric acid is a promising new drug for treating diabetes-related vascular endothelial damage. *In vitro* experiments have demonstrated that chicoric acid may protect against diabetes-induced endothelial dysfunction by activating the AMPK signaling pathway (Ma et al., 2021).

In the early stages of CVDs, damage to microvascular integrity and barrier function is considered the first step in diabetic vascular complications (Zhou et al., 2018). The new antidiabetic drug empagliflozin, a sodium-glucose cotransporter 2 (SGLT2) inhibitor, promotes urinary glucose excretion in a non-insulin-dependent manner (Zinman et al., 2015; Steven et al., 2017). Empagliflozin can maintain the cardiac microvascular integrity and barrier function in diabetic patients by improving diabetes-induced reductions in cadherins, increases in adhesion molecules, and capillary obstruction. Additionally, empagliflozin can reduce the diabetes-induced increase in the percentage of TUNEL (Terminal deoxynucleotidyl transferase dUTP nick end labeling assay, a method to detect apoptosis) positive cells (Zhou et al., 2018). Empagliflozin can inhibit excessive mitochondrial fission induced by long-term diabetes in cardiac microvascular endothelial cells (CMECs) (Figure 2B). Diabetes increases the translocation of Drp1 to fragmented mitochondria that carry more free fragments. However, in CMECs treated with empagliflozin or mdivi-1, the number of Drp1 foci on mitochondria is significantly reduced. The mechanism is that empagliflozin balances the phosphorylation of Drp1 at Ser616 and Ser637 through AMPK activation, ultimately leading to the failure of Drp1 recruitment to mitochondria (Zhou et al., 2018) (Figure 2C). Notably, FCCP inhibits the protective effect of empagliflozin on fission, leading to more mitochondria fragments labeled with Drp1 (Zhou et al., 2018). Diabetes significantly reduces the levels of fusion-related proteins, including Mfn1 and Mfn2. However, empagliflozin effectively upregulates fusion-related proteins while downregulating fission-related factors such as Fis1 and Mff (Zhou et al., 2018). Diabetic CMECs produce higher levels of mtROS and intracellular ROS, whereas treatment with mdivi-1 and empagliflozin limits this production (Zhou et al., 2018). Interestingly, activation of fission counteracts the ROS-scavenging effect of empagliflozin, suggesting that fission promotes oxidative stress induction (Zhou et al., 2018). Excess ROS hinders the cell cycle transition from the G0/G1 phase to the S phase, which is reversed by applying mtROS scavengers empagliflozin and mdivi-1 (Zhou et al., 2018). Additionally, preventing cellular senescence by neutralizing fission-induced ROS also improves vascular endothelial barrier function (Zhou et al., 2018). Reduced residual FITC-dextran content and increased TER (Transendothelial Electrical Resistance) values (Zhou et al., 2018). This indicates that empagliflozin has a beneficial effect on CMEC permeability and barrier function by inhibiting mitochondrial fission and subsequent oxidative stress (Zhou et al., 2018). Furthermore, empagliflozin inhibits oxidative stress-mediated upregulation of ICAM-1 and VCAM-1 while restoring eNOS phosphorylation at Ser1177 (Zhou et al., 2018). Additionally, empagliflozin can significantly prevent cardiac dysfunction, inhibit cardiac hypertrophy and fibrosis, and reduce glycogen deposition in cardiac tissue (Wang et al., 2022). The mechanism by which empagliflozin exerts its protective effects against diabetic cardiomyopathy (DCM) involves the activation of Nrf2 and its downstream antioxidant genes, improving mitochondrial function by inhibiting mitochondrial fission, and effectively suppressing oxidative stress.

mtROS may be excessively produced under iron overload conditions, leading to mitochondrial dysfunction and endothelial cells damage. As a therapeutic approach, luteolin can target mitochondria and protect HUVECs from iron overload-induced damage through the ROS/ADMA/DDAHⅡ/eNOS/NO pathway (Chen et al., 2022). The therapeutic compound isoliquiritigenin, similar to resveratrol, possesses strong antioxidant properties and cardiovascular protective effects (Chen et al., 2023). Research by Chen et al. indicates that isoliquiritigenin can inhibit mitochondrial ferroptosis through the PRDX2-MFN2-ACSl4 pathway, thereby effectively protecting the cardiac microvasculature in diabetic patients (Figure 2D) (Chen et al., 2023).

Targeted drug therapy is gradually maturing, with new mitochondrial-targeted drugs like MitoQ demonstrating activity in a rat model of cardiac ischemia-reperfusion injury and successfully administered orally to humans (Milagros Rocha and Victor, 2007). MitoQ is a mitochondria-targeted antioxidant formed by combining a derivative of coenzyme Q10 (CoQ10) with a triphenylphosphonium (TPP) cation (Murphy and Smith, 2000; Milagros Rocha and Victor, 2007). The TPP component enables MitoQ to selectively enter mitochondria, as the negative potential of the mitochondrial inner membrane attracts and accumulates positively charged TPP molecules (Figure 2E) (Milagros Rocha and Victor, 2007). Meanwhile, TPP is quite effective in reducing intracellular ROS, thus preventing cell death (Sheu et al., 2006; Murphy and Smith, 2007). Once accumulated within the mitochondria, MitoQ is adsorbed onto the surface of the inner membrane, where it is continuously converted into its antioxidant quinol form through complex II in the respiratory chain (Kelso et al., 2001). This form can neutralize ROS such as superoxide anions and hydrogen peroxide, thereby reducing oxidative stress (Milagros Rocha and Victor, 2007; Maroz et al., 2009). Studies also show that MitoQ can protect cells from peroxynitrite damage, though it is much less effective at eliminating hydrogen peroxide compared to other quinols (James et al., 2007). Additionally, it can prevent lipid peroxidation, a key pathway of mitochondrial oxidative damage, through the reduced form of coenzyme Q (ubiquinol) (Milagros Rocha and Victor, 2007).

## 3 Cerebrovascular diseases

### 3.1 Ischemic stroke

As research progresses, the role of mitochondria in cerebrovascular diseases is equally significant. The study by Rutkai et al. indicates that mitochondrial function in brain vasculature remains sustained for up to 48 h after transient middle cerebral artery occlusion in rats (Rutkai et al., 2014). This finding highlights the potential importance of endothelial cell mitochondria in the recovery of vascular function post-stroke, providing new perspectives for future stroke treatments.

Research by Deng et al. found that the δ-opioid receptor (δOR) agonist [D-ala2, D-leu5]-Enkephalin (DADLE) significantly enhances mitochondrial autophagy in brain microvascular endothelial cells (BMECs) by upregulating the expression of transient receptor potential vanilloid subtype 4 (TRPV4), thereby effectively reducing cell damage during ischemia/reperfusion (I/R) injury (Deng et al., 2024). The related mechanism of activating TRPV4 to promote vascular regeneration in helping ischemic stroke patients recover has been demonstrated in previous studies (Chen et al., 2018). Moreover, the protective effects of DADLE can be blocked by TRPV4 inhibitors or RNA interference, revealing the critical role of TRPV4 in DADLE-induced mitochondrial autophagy (Deng et al., 2024). The study also found that DADLE not only upregulates TRPV4 expression but also promotes mitochondrial autophagy by increasing calcium ion influx, thereby enhancing mitochondrial membrane potential and ATP synthesis, which in turn boosts cell viability (Deng et al., 2024). This study not only provides new insights into the mechanism of δOR agonists in protecting brain microvascular endothelial cells but also offers potential targets for developing new therapeutic strategies focusing on mitochondrial function and endothelial cell protection (Supplementary Figure S1).

Salvinorin A (SA), a κ-opioid receptor (KOR) agonist, has protective effects on mitochondrial function in brain vascular endothelial cells after ischemic stroke (Dong et al., 2019). The KOR is widely distributed in the human brain, with high expression in areas such as the cerebral cortex, hippocampus, and striatum (Mansour et al., 1995). Activation of KOR can significantly reduce neural damage and brain edema caused by ischemia/reperfusion, protecting the function of the blood-brain barrier (Yang et al., 2011; Chen et al., 2016). SA, a short-acting, highly selective KOR agonist, has unique advantages in ischemia/reperfusion injury due to its ability to easily cross the blood-brain barrier and its *in vivo* safety (Chen et al., 2016). By establishing a middle cerebral artery occlusion (MCAO) model in rats and implementing oxygen-glucose deprivation (OGD) in human brain microvascular endothelial cells (HBMECs), the study found that SA significantly reduces infarct volume and brain edema and improves the integrity of the blood-brain barrier (Dong et al., 2019). The mechanism primarily involves SA enhancing mitochondrial morphology and function, reducing oxidative stress, and decreasing cell apoptosis by activating the AMPK/Mfn2 signaling pathway (Dong et al., 2019). Furthermore, the activation of AMPK is a crucial step for SA to exert its protective effects, which is validated by using the AMPK inhibitor Compound C and siRNA technology (Dong et al., 2019). These findings provide new insights into the neuroprotective mechanisms of SA in brain ischemic injury and may offer potential therapeutic strategies for clinical treatment.

Studies have also found that the Active Fraction of Polyrhachis vicina (Roger) (AFPR) significantly reduces neurological damage and infarct volume in cerebral ischemia/reperfusion (CIR) rats by targeting Sirtuin (SIRT) 3-mediated mitophagy while promoting angiogenesis in the damaged area (Wei et al., 2023). *In vitro* experiments, using bEnd.3 cells (a mouse brain microvascular endothelial cell line) to simulate CIR conditions, it was found that AFPR activated the Pink1/Parkin signaling pathway, enhanced mitochondrial autophagy, reduced the accumulation of damaged mitochondria, and increased the deacetylation activity of SIRT3 by lowering the acetylation levels of FOXO3A (Wei et al., 2023). Additionally, AFPR-treated cells exhibited improved angiogenic capacity, including increased migration ability, promotion of tubular structure formation, and upregulation of vascular endothelial growth factor (VEGF)-A expression (Wei et al., 2023). These results suggest that AFPR has potential therapeutic value against cerebral ischemia-reperfusion injury by promoting endothelial cell mitophagy and improving angiogenesis (Supplementary Figure S2).

Zhou et al. revealed that Guhong Injection (GHI) protects the mitochondrial integrity of brain microvascular endothelial cells in a cerebral ischemia model (Zhou et al., 2021). The study demonstrated that GHI significantly reduces ischemia-induced cell apoptosis by activating the PI3K/AKT signaling pathway and by maintaining mitochondrial membrane potential, thereby inhibiting the amplification of apoptotic signals through reduced cytochrome c release (Zhou et al., 2021). Additionally, GHI increased the activity of superoxide dismutase (SOD) and decreased levels of lactate dehydrogenase (LDH), matrix metalloproteinase-9 (MMP-9), and malondialdehyde (MDA), indicating its positive effects against oxidative stress and cellular damage (Zhou et al., 2021). These findings provide new insights into the application of GHI in the treatment of cerebral ischemia, particularly in protecting mitochondrial function in brain endothelial cells.

Dave et al. demonstrated that utilizing medium to large-sized extracellular vesicles (m/lEVs) containing mitochondria, along with exogenous 27 kDa heat shock protein (HSP27), significantly improves mitochondrial function and survival in brain endothelial cells while protecting tight junction integrity (Dave et al., 2023). This approach provides a novel therapeutic strategy for protecting the blood-brain barrier following ischemic stroke. Recent research advances indicate that extracellular vesicles (EVs), particularly microvesicles (MVs), play a crucial role in regulating endothelial cell mitochondrial function. D’Souza et al. first demonstrated that MVs derived from the human brain endothelial cell line hCMEC/D3 can transfer polarized mitochondria to recipient brain endothelial cells, as well as neurons in mouse cortical and hippocampal slices (D’Souza et al., 2021). This mitochondrial transfer significantly increased ATP levels in recipient cells under OGD conditions, enhancing cell survival (D’Souza et al., 2021). Furthermore, the study found that MVs are more effective than exosomes (EXOs) in enhancing mitochondrial function in recipient cells (D’Souza et al., 2021). These findings provide new scientific evidence for the potential use of EVs, particularly MVs, as therapeutic tools to enhance mitochondrial function and improve the survival of brain endothelial cells under ischemic conditions.

In addition, research on using vesicles for cerebral ischemia treatment revealed that EVs derived from mouse brain endothelial cells (mBEC), which contain mitochondria, are more effective than those derived from human brain endothelial cells (hBEC) in enhancing mitochondrial function in ischemic mouse brain endothelial cells (Dave et al., 2024). These mBEC-EVs significantly increased cellular ATP levels by boosting oxygen consumption, reduced the infarct volume in a mouse middle cerebral artery occlusion (MCAo) model, and improved behavioral recovery (Dave et al., 2024). The results highlight the potential advantages of isotypic EVs in treating ischemic stroke, particularly when the donor species is homologous to the recipient species, which may enhance therapeutic efficacy. This supports further exploration of isotypic EVs in preclinical studies.

In addition, studies on stem cell therapy in cerebral ischemia have revealed that mitochondrial transfer can serve as a novel therapeutic mechanism. Research indicates that mesenchymal stem cells (MSCs) can transfer mitochondria to damaged vascular endothelial cells and neurons through tunneling nanotubes (TNTs) or mitochondria-containing MVs, thereby restoring their metabolic function and enhancing their survival (Huang et al., 2020). Particularly in hypoxia-reoxygenation injury models, mitochondrial transfer derived from endothelial progenitor cells (EPCs) has been shown to enhance endothelial permeability, mitochondrial biogenesis, mitochondrial DNA (mtDNA) copy number, and intracellular ATP levels, potentially enhancing endothelial cell tightness by upregulating specific protective genes (Huang et al., 2020). Furthermore, the efficiency and effectiveness of mitochondrial transfer are regulated by various signaling molecules, including Miro1 and Cx43, which are involved in the formation of TNTs and the regulation of gap junctions, respectively (Huang et al., 2020). These findings provide new insights into utilizing stem cell therapy for ischemic brain injury and lay the foundation for the development of future therapeutic strategies.

Panickar et al. demonstrated that specific polyphenolic compounds, including Cinnamtannin D1 from cinnamon, green tea extract, and resveratrol, can significantly alleviate ischemia-induced mitochondrial dysfunction in endothelial cells *in vitro* (Panickar et al., 2015). These polyphenols reduce mtROS production, maintain the stability of mitochondrial membrane potential (ΔΨm), and decrease intracellular calcium concentration ([Ca^2+^]i), effectively preventing endothelial cell swelling caused by OGD (Panickar et al., 2015). Additionally, the study found that MCP-1 exacerbates OGD-induced cell swelling, while polyphenolic intervention mitigates this effect (Panickar et al., 2015). These findings provide important insights into the role of polyphenolic compounds in protecting endothelial cell mitochondria from ischemic damage and offer a scientific basis for developing potential therapeutic strategies for cerebral ischemia.

Estrogen (E2) exerts a protective effect on brain endothelial cell viability and mitochondrial function following *in vitro* ischemic injury through an estrogen receptor (ER)-mediated mechanism. The study found that long-term (24 h or 48 h) pretreatment with 10 nmol/L E2 could prevent cell death induced by OGD/R, while short-term (0.5 h or 12 h) pretreatment was ineffective (Guo et al., 2010). The protective effect of E2 could be mimicked by the ERα-selective agonist PPT, but not by the ERβ-selective agonist DPN (Guo et al., 2010). This indicates that in this mechanism, E2 primarily exerts its protective effect through ERα. Furthermore, E2 significantly reduced the production of mitochondrial superoxide anions and maintained mitochondrial membrane potential and ATP levels during the early stages of OGD/reperfusion (Guo et al., 2010). All effects of E2 could be blocked by the ER antagonist ICI-182,780 (Guo et al., 2010). These results indicate that E2 can protect mitochondrial function through an ER-mediated mechanism, thereby providing protection to brain endothelial cells from ischemic injury.

Studies have shown that laminar shear stress (LS) significantly enhances the survival rate of BMECs under ischemic conditions by regulating the expression of Tie-2, Bcl-2, and Akt, which are associated with cell membrane, mitochondrial, and nuclear functions (Tian et al., 2013). Specifically, the maintenance of mitochondrial function is achieved through the Bcl-2 protein, which protects the integrity of the mitochondrial membrane by regulating cytochrome c release, counteracting energy metabolism imbalance, maintaining membrane permeability, and preventing the collapse of mitochondrial membrane potential (Petit et al., 1996). This study highlights the importance of restoring cerebral blood flow and promoting LS in the treatment of ischemic stroke, providing new perspectives for future therapeutic strategies.

The above studies indicate that research on mitochondrial function, metabolism, and mechanisms holds promise for playing a significant role in the treatment of cerebrovascular diseases.

### 3.2 Alzheimer’s disease

In the pathophysiology of AD, mitochondrial dysfunction in endothelial cells plays a crucial role. Research by Gjumrakch Aliev et al. indicates that oxidative stress damages cerebral vascular endothelial cells by increasing levels of ROS, which in turn affects the integrity and function of the endothelial barrier (Aliev et al., 2002). Specifically, ROS-induced nitric oxide (NO) degradation weakens the vasodilatory effect of NO and promotes endothelin-1 (ET-1)-mediated vasoconstriction, potentially leading to chronic cerebral hypoperfusion and the development of AD (Aliev et al., 2002). Additionally, Aβ deposition around cerebral blood vessels may exacerbate oxidative stress-induced vascular damage (Aliev et al., 2002). The study also found that mitochondrial DNA depletion is associated with oxidative damage, which is particularly pronounced in damaged neurons of AD patients (Aliev et al., 2002). Therefore, protecting mitochondrial function and reducing oxidative stress may offer new strategies for treating AD. Mitochondrial dysfunction contributes to the onset of neurodegenerative diseases. The antioxidant function of mitochondria gradually diminishes with aging, leading to increased production of free radicals (Lyros et al., 2014). These free radicals further impair mitochondrial function, damaging ATP production and resulting in further free radical generation (Schulz et al., 1997). Mitochondrial dysfunction and free radical production create a vicious cycle (Pang et al., 2024). The generated free radicals can induce the production of Aβ, leading to the development of AD (Lyros et al., 2014) (Supplementary Figure S3). Mitochondrial damage is a significant feature of AD and a potential therapeutic target (Liu et al., 2021). Although VEGF has been shown to improve mitochondrial function in endothelial cells by activating the mTOR signaling pathway (Guo et al., 2017), whether VEGF can alleviate Aβ-induced mitochondrial damage is unknown. Liu et al. demonstrated that VEGF promotes mitochondrial biogenesis by activating the PGC-1α-NRF1/2-TFAM pathway, which not only improves mitochondrial function but also increases mitochondrial quantity (Supplementary Figure S4) (Liu et al., 2021). Meanwhile, this significantly enhances spatial learning and memory in AD model mice, reduces Aβ levels, improves cell viability, and decreases ROS production, thereby positively impacting mitochondrial structure and function (Liu et al., 2021).

As a progressive neurodegenerative disease, one of the early pathological events in AD is cerebrovascular dysfunction, which is particularly prominent in AD and mixed dementia (Iadecola, 2010; Corriveau et al., 2016). Cerebral amyloid angiopathy (CAA) is a common pathological feature in AD, characterized by the deposition of Aβ around cerebral blood vessels, leading to vascular dysfunction, including hemodynamic abnormalities and blood-brain barrier (BBB) leakage (Ghiso et al., 2014). Endothelial mitochondrial dysfunction plays a crucial role in the pathogenesis of AD and CAA, affecting energy metabolism, the production of ROS, NO, and reactive nitrogen species (RNS), as well as calcium (Ca^2+^) signaling and apoptosis between the endoplasmic reticulum and mitochondria (Parodi-Rullán et al., 2019). Aβ negatively impacts mitochondrial function in BMECs, resulting in decreased ATP production and increased oxidative stress, which in turn affects neurovascular function (Parodi-Rullán et al., 2019). Potential therapeutic strategies targeting mitochondrial dysfunction, including both pharmacological and non-pharmacological approaches, may help prevent or delay cerebrovascular failure in AD and CAA.

When exploring the role of endothelial cell mitochondria, their central function in the neurovascular unit cannot be overlooked. Particularly in the pathological processes of AD and CAA, the interaction between mitochondrial damage in endothelial cells and vascular inflammation as well as neurodegeneration is crucial (Parodi-Rullán et al., 2019). The studies by Rebecca provide an in-depth analysis of this interaction, revealing how Aβ peptides, by binding to receptors on endothelial cells, trigger excessive production of ROS in mitochondria, leading to the loss of mitochondrial membrane potential and the release of cytochrome C (Supplementary Figure S5) (Parodi-Rullán et al., 2021). These events activate inflammasomes, particularly the NLRP3 inflammasome, which promotes the production of inflammatory mediators and activation of endothelial cells (Parodi-Rullán et al., 2021). Additionally, the study highlights the potential role of mitochondrial DNA (mtDNA) as damage-associated molecular patterns (DAMPs) in the inflammatory response, and the potential therapeutic value of mitochondrial-targeted antioxidants such as MitoQ and SS-31 peptides in alleviating vascular inflammation and protecting the neurovascular unit (Supplementary Figure S6) (Parodi-Rullán et al., 2021). These findings not only enhance our understanding of the mechanisms of neurovascular damage in AD and CAA from a mitochondrial perspective but also provide a scientific basis for the development of new therapeutic strategies.

The research by Lyros et al. emphasizes the critical role of mitochondria in AD, noting that mitochondria are not only sites of energy production but also important sensors in maintaining the balance of the brain microenvironment (Lyros et al., 2014). Stimulation by Aβ leads to mitochondrial dysfunction and activation of programmed cell death in endothelial cells, suggesting that Aβ synthesis may be an upstream event in endothelial dysfunction in AD (Xu et al., 2001). Additionally, mitochondrial dysfunction leads to an increase in ROS, which promotes the production of Aβ both *in vivo* and *in vitro* (Leuner et al., 2012). This may create a vicious cycle involving mitochondrial dysfunction and Aβ. Meanwhile, Aliev et al. highlighted the significance of addressing mitochondrial dysfunction in AD treatment strategies (Aliev et al., 2009). They discovered that oxidative stress caused by chronic hypoperfusion is a major factor inducing mitochondrial failure, a process that affects not only neurons but also damages vascular endothelial cells, leading to decreased blood-brain barrier function (Aliev et al., 2009). Additionally, the coexistence of mitochondrial DNA loss and oxidative damage markers in AD further confirms the central role of mitochondria in vascular pathology (Aliev et al., 2009).

Therefore, exploring the efficacy and potential mechanisms of mitochondrial function in AD is promising for developing more effective therapeutic strategies to improve patient outcomes and enhance quality of life.

## 4 Conclusion and perspectives

As flat cells lining the blood vessels, endothelial cells constitute a physical and biological barrier between the blood and tissues. They play an indispensable role in regulating vascular tone by releasing various substances that either dilate or constrict blood vessels. Additionally, endothelial cells maintain the structural integrity of the vasculature by producing extracellular matrix components and regulating cell adhesion and proliferation. As the first line of defense for vascular health, their dysfunction is closely linked to cardiovascular and cerebrovascular diseases. Mitochondria, the powerhouses of cells, meet cellular energy demands by producing ATP from oxygen and glucose. Beyond energy production, mitochondria regulate several key biological processes in endothelial cells, including redox signaling, cellular aging, calcium homeostasis, angiogenesis, apoptosis, and inflammation (Chang et al., 2021). Additionally, mitochondria in endothelial cells are considered central oxygen sensors within the vascular system, acting as local environmental sensors (Chang et al., 2021). Current clinical and basic research points to mitochondrial dysfunction as a significant driver of endothelial damage and CVDs. This review provides a comprehensive overview of the pathogenesis of endothelial injury in CVDs, cerebrovascular diseases, and AD. By elaborating on the roles and mechanisms of mitochondria in these conditions, the review aims to develop more effective therapeutic strategies. It also offers potential treatment strategies for cardiovascular and cerebrovascular diseases, including the use of clinical drugs, antioxidants, stem cell therapy, and specific polyphenolic substances, to provide new insights and treatment approaches for vascular injury. This information is intended to offer references for improving patient prognosis and enhancing quality of life in clinical settings.

Future research in vascular diseases should delve into the molecular and mechanistic links between mitochondrial abnormalities and endothelial damage, and explore how mitochondrial function can be modulated to prevent and treat CVDs. New therapeutic strategies, including drugs, antioxidants, stem cell therapy, and specific polyphenolic substances, may offer new directions and solutions for the prevention and treatment of CVDs by regulating mitochondrial dynamics, promoting mitochondrial autophagy, and inhibiting ROS production. These strategies could also provide insights and approaches for using improved mitochondrial function to prevent or treat other clinical conditions.
