# Supplementary material for: Advances in pathogenesis and treatment of vascular endothelial injury-related diseases mediated by mitochondrial abnormality
Source: Front Pharmacol. 2024 Aug 30;15:1422686. doi: 10.3389/fphar.2024.1422686 (PMC11394189; doi:10.3389/fphar.2024.1422686)
Supplement: Supplementary file 1 [file DataSheet1.docx]

**Supplementary Material**

**Advances in Pathogenesis and Treatment of Vascular Endothelial Injury-Related Diseases Mediated by Mitochondrial Abnormality**

Boxian Pang^1,4†^; Guangtong Dong^1†^; Tieliang Pang^2†^; Xinyao Sun^1†^; Xin Liu^3^; Yifeng Nie^4*^; Xing Chang^5*^

1. Beijing University of Chinese Medicine, Beijing, China
2. Beijing Anding hospital, Capital Medical University, Beijing, China
3. Bioscience Department, University of Nottingham, Nottingham, UK
4. CAS Center for Excellence in Nanoscience, National Center for Nanoscience and Technology, Beijing, China.
5. Guang’anmen Hospital, China Academy of Chinese Medical Sciences, 5 Beixiange, Xicheng District, Beijing, China

***Corresponding Author:**

Yifeng Nie: nieyf@nanoctr.cn

Xing Chang: [xingchang_tcm@outlook.com](mailto:xingchang_tcm@outlook.com)

**†These authors have contributed equally to this work and share first authorship**

**
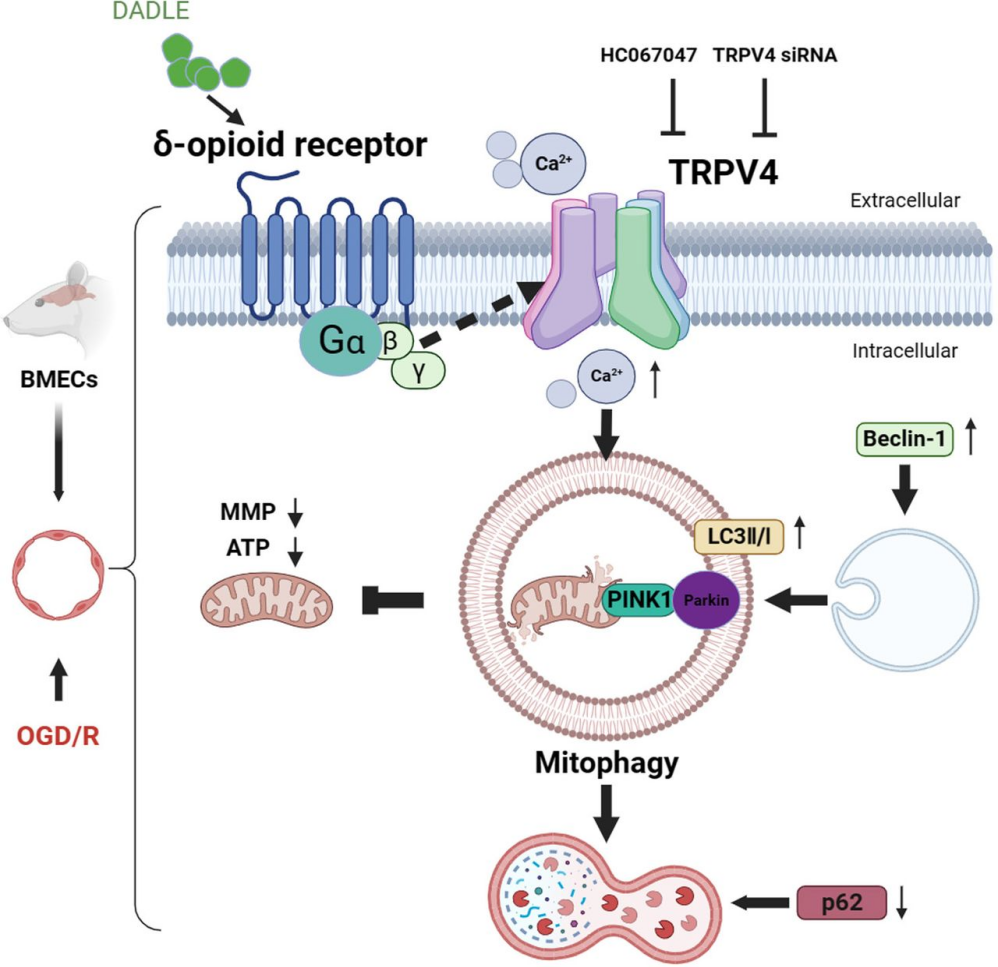
**

# Figure S1. The mechanism of Delta opioid peptide [D-ala2, D-leu5]-Enkephalin enhances mitophagy via TRPV4 to relieve ischemia injury in brain microvascular endothelial cells. BMECs: brain microvascular endothelial cells; DADLE:[D-ala2, D-leu5]-Enkephalin; I/R: ischaemia/reperfusion; OGD/R: oxygen-glucose deprivation/reoxygenation; TRPV4:transient receptor potential subfamily V member 4. Copyright 2022, Reprinted with from permission from BMJ Publishing Group Ltd. (Deng et al., 2024).

**
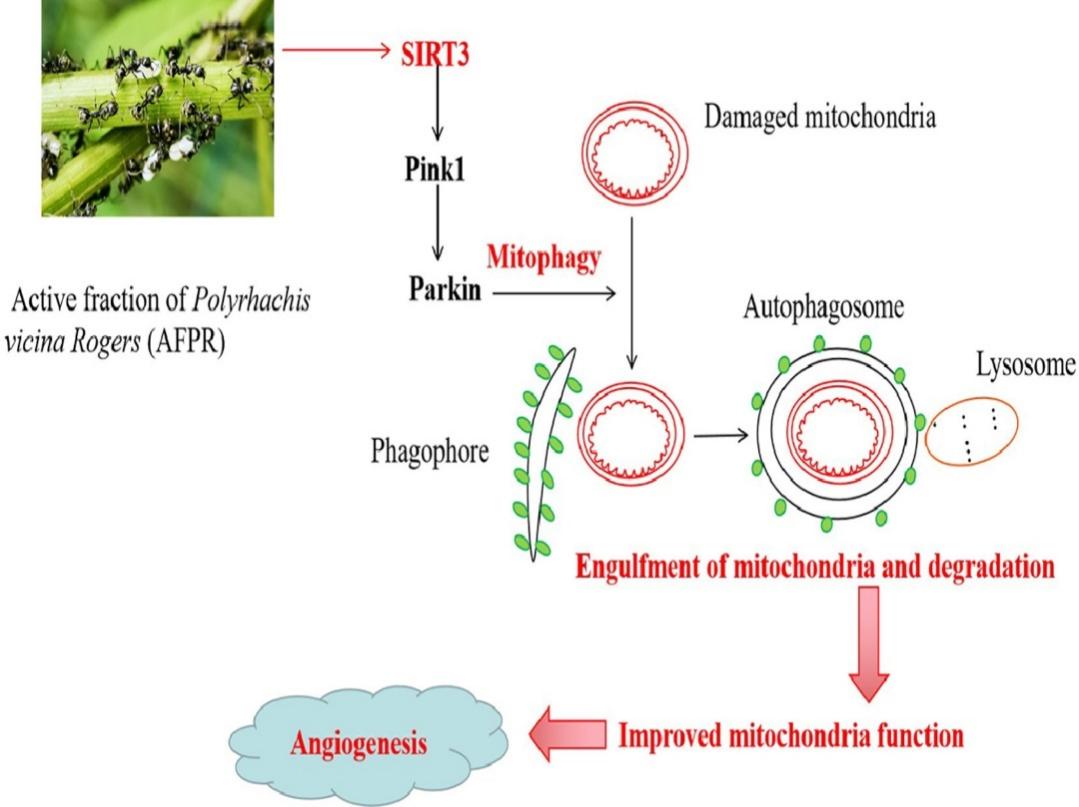
**

Figure S2. Molecular mechanism of action of AFPR against CIR Ischemia induced endothelial mitochondria dysfunction in stroke. Ischemia can lead to mitochondrial dysfunction in endothelial cells, where impaired mitophagy results in the accumulation of damaged mitochondria, potentially exacerbating endothelial dysfunction. AFPR specifically targets the function of SIRT3, enhancing its deacetylase activity. This increase in SIRT3 activity helps to maintain a low acetylation level of the transcription factor FOXO3A. With reduced acetylation, FOXO3A is more effective in promoting the transcription of the gene encoding PINK1, a protein that plays a crucial role in the mitophagy process. The activation of the PINK1/Parkin pathway facilitates the selective removal of damaged mitochondria through mitophagy. Mitophagy is characterized by the engulfment of impaired mitochondria by autophagosomes, followed by their degradation within lysosomes. This process effectively clears abnormal mitochondria, aiding in the restoration of mitochondrial homeostasis. By doing so, it helps to preserve endothelial function following ischemia and reperfusion events, thereby mitigating the impact of cerebral ischemia. Copyright 2023, Reprinted with from permission from Elsevier B.V. (Wei et al., 2023)


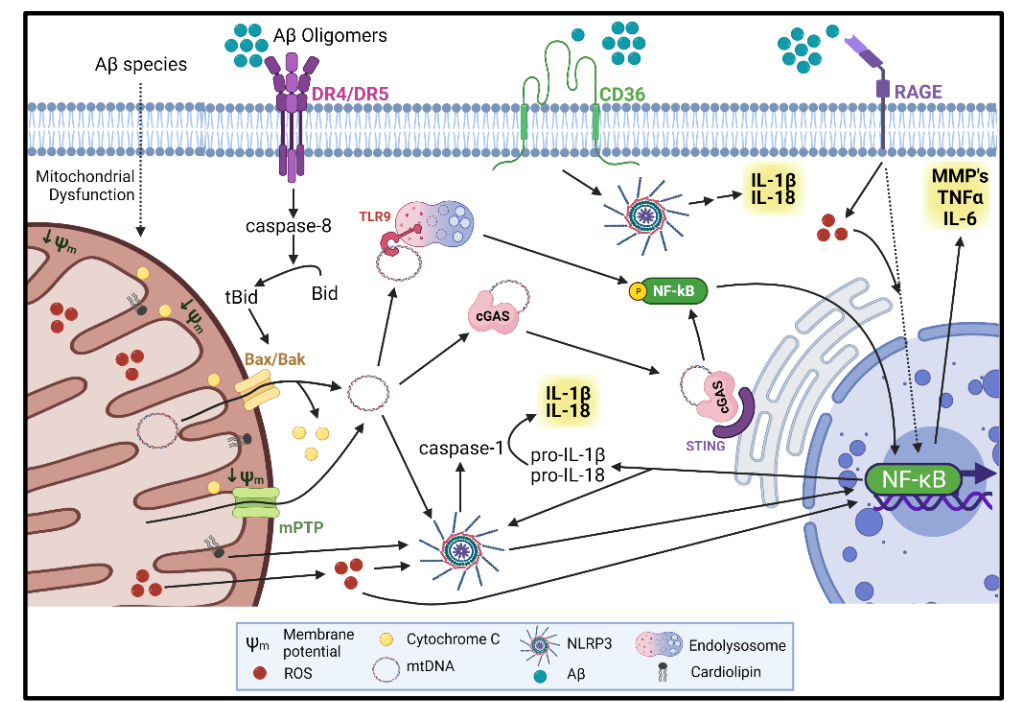


Figure S3. Amyloid-β (Aβ) peptides trigger the release of mitochondrial damage-associated molecular patterns (DAMPs) in cerebral endothelial cells (ECs), which in turn activate the vascular system. Aβ induces mitochondrial dysfunction through mechanisms that include binding to and activating TRAIL Death Receptors (DRs), thereby initiating both the extrinsic and intrinsic apoptotic pathways.Aβ has been observed to cause a loss of mitochondrial membrane potential (Ψm), an increase in mitochondrial reactive oxygen species (mtROS) production, and the permeabilization of mitochondrial membranes. This permeabilization results in the release of cytochrome C and mitochondrial DNA (mtDNA) into the cytoplasm of endothelial cells. The elevated levels of mtROS activate the NLRP3 inflammasome, leading to the activation of caspase-1 and NFκB, which in turn promote the release of pro-inflammatory cytokines IL-1β and IL-18. The permeabilization of both the inner mitochondrial membrane (IMM) and the outer mitochondrial membrane (OMM) allows mtDNA to be released into the cytoplasm. The presence of double-stranded DNA (dsDNA) then activates the NLRP3 inflammasome, toll-like receptor 9 (TLR9) within the endolysosomal compartment, and the cGAS/STING pathway on the cytosol and endoplasmic reticulum (ER) membrane. These activations lead to the stimulation of NFκB and the upregulation of additional pro-inflammatory cytokines.Furthermore, Aβ can directly activate the NLRP3 inflammasome by binding to the CD36 membrane receptor or increase ROS production through the RAGE receptor. This leads to a significant increase in pro-inflammatory cytokines, which can disrupt the blood-brain barrier (BBB). The disruption is facilitated by the activation of matrix metalloproteinases (MMPs), downregulation of tight junction (TJ) proteins, endothelial cell activation, and the expression of cell adhesion molecules. Copyright 2021, Reprinted with from permission from MDPI. (Parodi-Rullán et al., 2021)


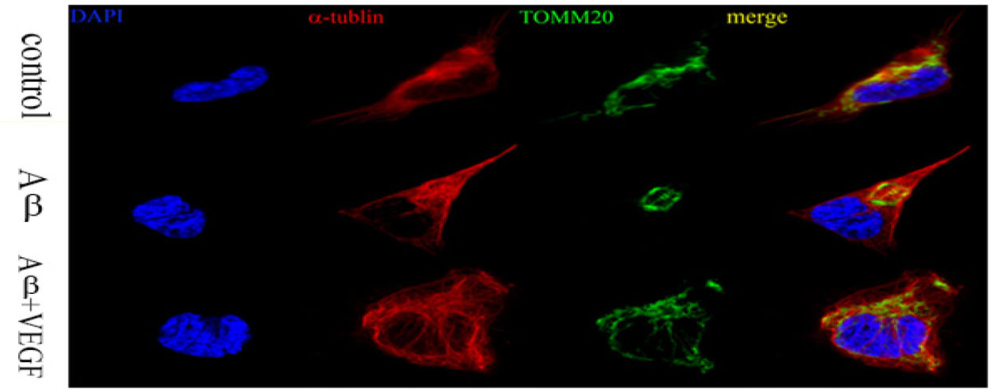


Figure S4.Vascular endothelial growth factor alleviates mitochondrial dysfunction and protects mitochondrial structure, function and number. Representative confocal immunofluorescent images. Blue fluorescence indicates cell nucleus, red cytoskeleton and green mitochondria. Copyright 2020, Reprinted with from permission from Taylor & Francis. (Liu et al., 2021)

.


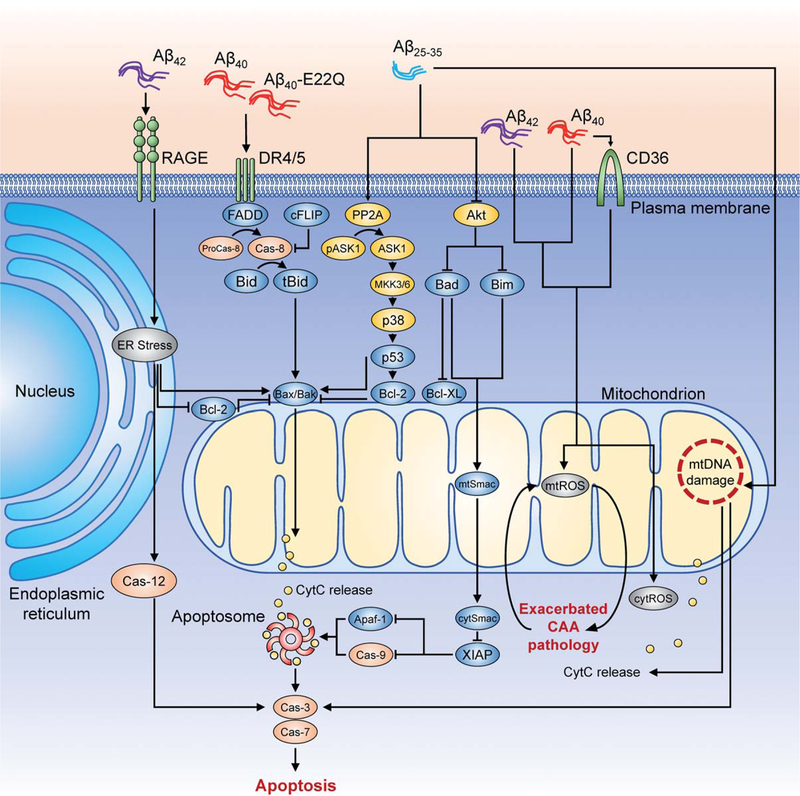


Figure S5. Amyloid-induced apoptotic pathways involving the mitochondria in cerebral endothelial cells. Amyloid peptides, such as Aβ40 and Aβ42, are implicated in triggering apoptosis in cerebral endothelial cells (CECs) through mitochondrial pathways. The depicted mechanism illustrates how Aβ peptides interact with membrane receptors, including Receptor for Advanced Glycation End-products (RAGE), CD36, and Death Receptors 4/5 (DR4/5). These interactions initiate both mitochondria-dependent and -independent activation of caspases (Cas), which are key enzymes in the execution of apoptosis.The presence of Aβ peptides leads to an increase in reactive oxygen species (ROS) within both the mitochondrial matrix and the cytosol, as well as the release of cytochrome c (CytC) from the mitochondria into the cytosol. This release is a critical step in the intrinsic apoptotic pathway, as it facilitates the formation of the apoptosome complex, which in turn activates caspase-9 and subsequently effector caspases.Furthermore, Aβ peptides have been shown to activate the PP2A-mediated signaling cascade, a process that can counteract the survival-promoting effects of the Akt pathway. The inhibition of Akt results in the activation of pro-apoptotic proteins both in the cytosol and within the mitochondria, amplifying the apoptotic signal.Additionally, Aβ peptides can cause direct damage to mitochondrial DNA (mtDNA), which impairs mitochondrial function and contributes to the apoptotic process in CECs. This DNA damage further exacerbates the loss of mitochondrial integrity and the subsequent activation of apoptosis, highlighting the multifaceted role of Aβ peptides in the demise of cerebral endothelial cells.Copyright 2019, Reprinted with from permission from NIH Public Access. (Parodi-Rullán et al., 2019)


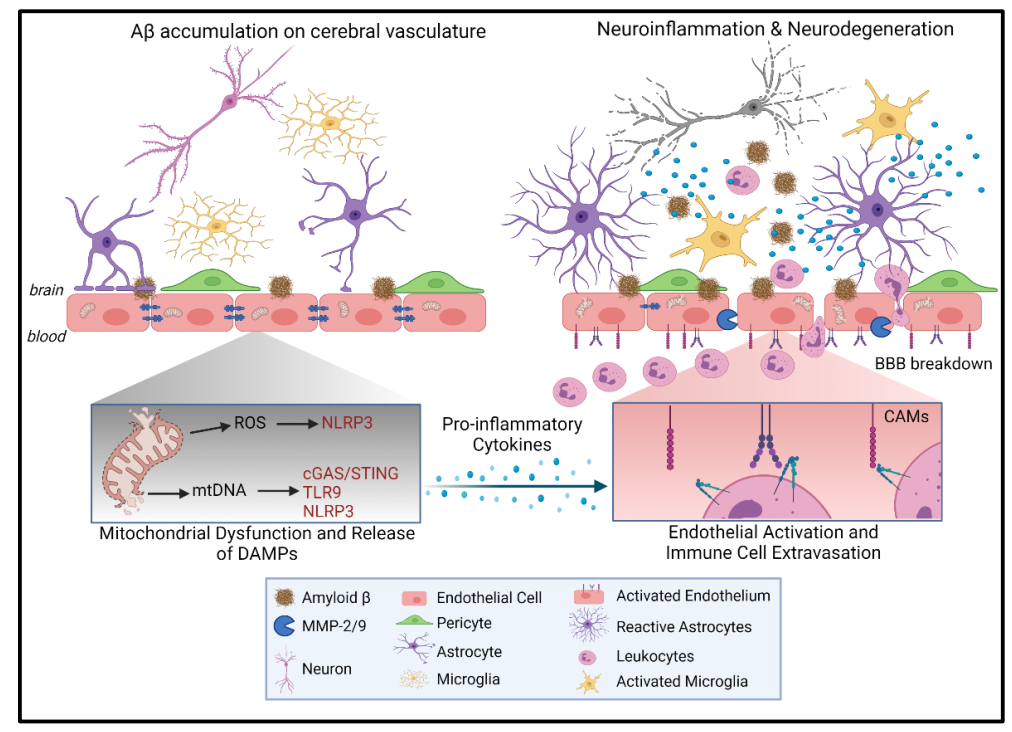


Figure S6. The Aβ-mediated release of mitochondrial DAMPs, such as mitochondrial reactive oxygen species (mtROS) and mitochondrial DNA (mtDNA), from brain endothelial cells (EC) induces a perivascular inflammatory response. This in turn leads to activation of EC, which drives expression of cell adhesion molecules (CAM), leading to cerebral vasculature cell damage. Copyright 2021, Reprinted with from permission from MDPI. (Parodi-Rullán et al., 2021).

**Reference**

Deng, Z., Chen, X., Zhang, R., Kong, L., Fang, Y., Guo, J., et al. (2024). Delta opioid peptide [D-ala2, D-leu5]-Enkephalin’s ability to enhance mitophagy via TRPV4 to relieve ischemia/reperfusion injury in brain microvascular endothelial cells. Stroke Vasc. Neurol., 003080. doi:10.1136/svn-2023-003080

Liu, X., Chu, B., Jin, S., Li, M., Xu, Y., Yang, H., et al. (2021). Vascular endothelial growth factor alleviates mitochondrial dysfunction and suppression of mitochondrial biogenesis in models of Alzheimer’s disease. Int. J. Neurosci. 131, 154–162. doi:10.1080/00207454.2020.1733564

Parodi-Rullán, R., Sone, J. Y., and Fossati, S. (2019). Endothelial mitochondrial dysfunction in cerebral amyloid angiopathy and Alzheimer’s disease. JAD 72,1019–1039. doi:10.3233/JAD-190357

Parodi-Rullán, R. M., Javadov, S., and Fossati, S. (2021). Dissecting the crosstalk between endothelial mitochondrial damage, vascular inflammation, and neurodegeneration in cerebral amyloid angiopathy and Alzheimer’s disease. Cells 10, 2903. doi:10.3390/cells10112903

Wei, J., Xie, J., He, J., Li, D., Wei, D., Li, Y., et al. (2023). Active fraction of Polyrhachis vicina (Roger) alleviated cerebral ischemia/reperfusion injury by targeting SIRT3-mediated mitophagy and angiogenesis. Phytomedicine 121, 155104. doi:10.1016/j.phymed.2023.155104
